# Involvement of Peripheral Opioid Receptors in the Realization of Food Motivation Into Eating Behavior

**DOI:** 10.3389/fnbeh.2020.600920

**Published:** 2021-01-12

**Authors:** Sergey Sudakov, Natalia Bogdanova

**Affiliations:** Laboratory of Physiology of Reinforcement, P.K. Anokhin Research Institute of Normal Physiology, Moscow, Russia

**Keywords:** metabolism, motor activity, operant eating behavior, gastric opioid receptors, food motivation, DAMGO, DADLE

## Abstract

The involvement of peripheral opioid receptors in the mechanisms of eating behavior is still unclear. The aim of this work was to study the role of peripheral, predominantly gastric mu and delta opioid receptors in the realization of food motivation in conditions of different energy costs for eating behavior. Experiments were performed under a between-sessions progressive ratio schedule of reinforcement in food-deprived rats. The level of food motivation was calculated using a self-developed method. Food intake, motor activity, and metabolic rate were recorded in fed and hungry animals. Results showed that intragastric administration of the mu opioid receptor agonist DAMGO led to an increase in the level of food motivation in the light variant of operant feeding behaviors. Food consumption did not change. At high costs for feeding behavior, the administration of DAMGO did not alter food motivation; however, food consumption and motor activity were reduced. Intragastric administration of the delta opioid receptor agonist DADLE did not lead to changes in the level of food motivation and physical activity, but inhibition of feeding behavior was observed in all reinforcement schedules. Three regulatory pathways of eating behavior in difficult food conditions by peripheral, predominantly gastric opioid receptors are hypothesized: environmental-inhibitory afferentations and suppression of the realization of food motivation into behavior; homeostatic-inhibitory action on food motivation; and rewarding-suppression of the anticipatory reinforcement.

## Introduction

The endogenous opioid system plays an important role in the organization of feeding motivation and eating behavior (Holtzman, 1979; Morley et al., 1983; Gosnell et al., 1986; Drewnowski et al., 1992; Selleck et al., 2018; Bodnar, 2019). It has been shown that activation of mu opioid receptors in a number of brain structures leads to activation of eating behavior in rodents (Zhang et al., 1998), but inhibits food intake in birds (Bungo et al., 2004). Activation of delta opioid receptors of the brain, as a rule, leads to hyperphagia (Majeed et al., 1986; Bakshi and Kelley, 1993). However, the role of delta opioid signaling in modulating feeding is not fully understood, as administration of delta opioid antagonists in the nucleus accumbens also causes an increase in food consumption (Kelley et al., 1996). The effect of opioids on food motivation is difficult to evaluate since most studies rely on measurements of the amount of food eaten. Classical breakpoint can be used to gauge the level of motivation and this method incorporates within- and between-sessions progressive ratio schedules and is defined as the last fixed ratio response in the increasing fixed ratio progression. For example, in rodent studies, this can be both the number of the lever presses (Spear and Katz, 1991) and the holding time of the pressed lever (Gulotta and Byrne, 2015). However, this approach is most often used to assess the severity of the reinforcer effect and does not allow separation of such systemic mechanisms of eating behavior as motivation, anticipation, and the actual behavioral act of eating. It was shown that the opioid system of the brain mainly takes part in the anticipatory component of eating behavior and does not participate in the mechanisms of motivational and consummative components (Barbano and Cador, 2006). However, for such a study, a separate series of experiments are needed, using different methods to study the corresponding components of eating behavior. The method we developed (Sudakov et al., 2015) made it possible, using the same animals in the same environment, to study the effect of opioid agonists both on food motivation and on its implementation in nutritional behavior.

The endogenous opioid system consists of central and peripheral divisions which share the same receptors, endogenous peptide ligands, and enzymes for their formation and degradation. The divisions are separated by a blood-brain barrier that restricts opioid peptides. A large number of peripheral opioid receptors are located in the gastrointestinal tract (Holzer, 2009). Their activation can take place when eating certain foods which contain substances with opioid activity such as casomorphin produced from milk casein (Brantl et al., 1979, 1982), exorphins and gliadorphine from gluten (Fukudome and Yoshikawa, 1993; Takahashi et al., 2000), soymorphin from soybean (Yamada et al., 2012), and rubiscolin from spinach (Yang et al., 2001). Theoretically, the formation of other peptides with opioid activity from food protein products under the action of pepsin is possible. Under normal conditions, in an adult mammal, opioid peptides from the gastrointestinal tract do not penetrate into the central nervous system. Opioid receptors located in the wall of the stomach and intestines can bind to these opioid ligands to induce local and behavioral effects (Dubynin et al., 2004, 2007; Belyaeva et al., 2008; Yoshikawa, 2015). Thus, orally ingested soymorphin has been found to suppress food intake by activating mu opioid receptors in the gastrointestinal tract (Kaneko et al., 2010). Orally administered rubiscolin-6 suppressed high-fat diet intake (Kaneko et al., 2014) and enhanced memory consolidation in a step-through type passive avoidance test (Yang et al., 2003). These effects were blocked by centrally administered naltrindole, an antagonist for the delta receptor.

In our previous studies, it was shown that activation of mu opioid receptors of the gastrointestinal tract leads to a decrease in the intercellular content of beta-endorphin, as well as to a decrease in the density and affinity of mu opioid receptors in the brain (Proskuryakova et al., 2009; Sudakov et al., 2010). The administration of opioid peptides into the stomach of rats caused a change in the level of motor activity, anxiety, and pain sensitivity (Alexeeva et al., 2012; Sudakov et al., 2017). Based on the obtained data, the hypothesis of reciprocal interaction of the peripheral and central parts of the endogenous opioid system was formulated (Sudakov and Trigub, 2008). This hypothesis implies that activation of peripheral, and gastric in particular, opioid receptors will lead to suppression of the central part of the endogenous opioid system (Sudakov, 2019). Thus, the effects on peripheral opioid receptors, altering the activity of central opioid mechanisms, could lead to some changes in eating behavior.

In our preliminary experiments we found that activation of peripheral, predominantly gastric opioid receptors may influence the operant feeding behavior in rat (Bogdanova et al., 2015). Other information on the participation of gastric opioid receptors in the mechanisms of regulation of eating behavior is currently not available. The aim of this work was to study the role of mu and delta opioid receptors on the implementation of feeding motivation into eating behavior in varying conditions of energy costs for this behavior. The objectives of the study were to examine the effect of intragastric administration of peptide agonists of mu and delta opioid receptors on: (I) food intake, metabolic rate, and motor activity in conditions of free access to food in home cages; and (II) food intake, the level of food motivation, and metabolism in test cages where conditions of instrumental feeding behavior were regulated under a between-sessions progressive ratio schedule of reinforcement.

## Materials and Methods

### Animals

**Figure 1:**
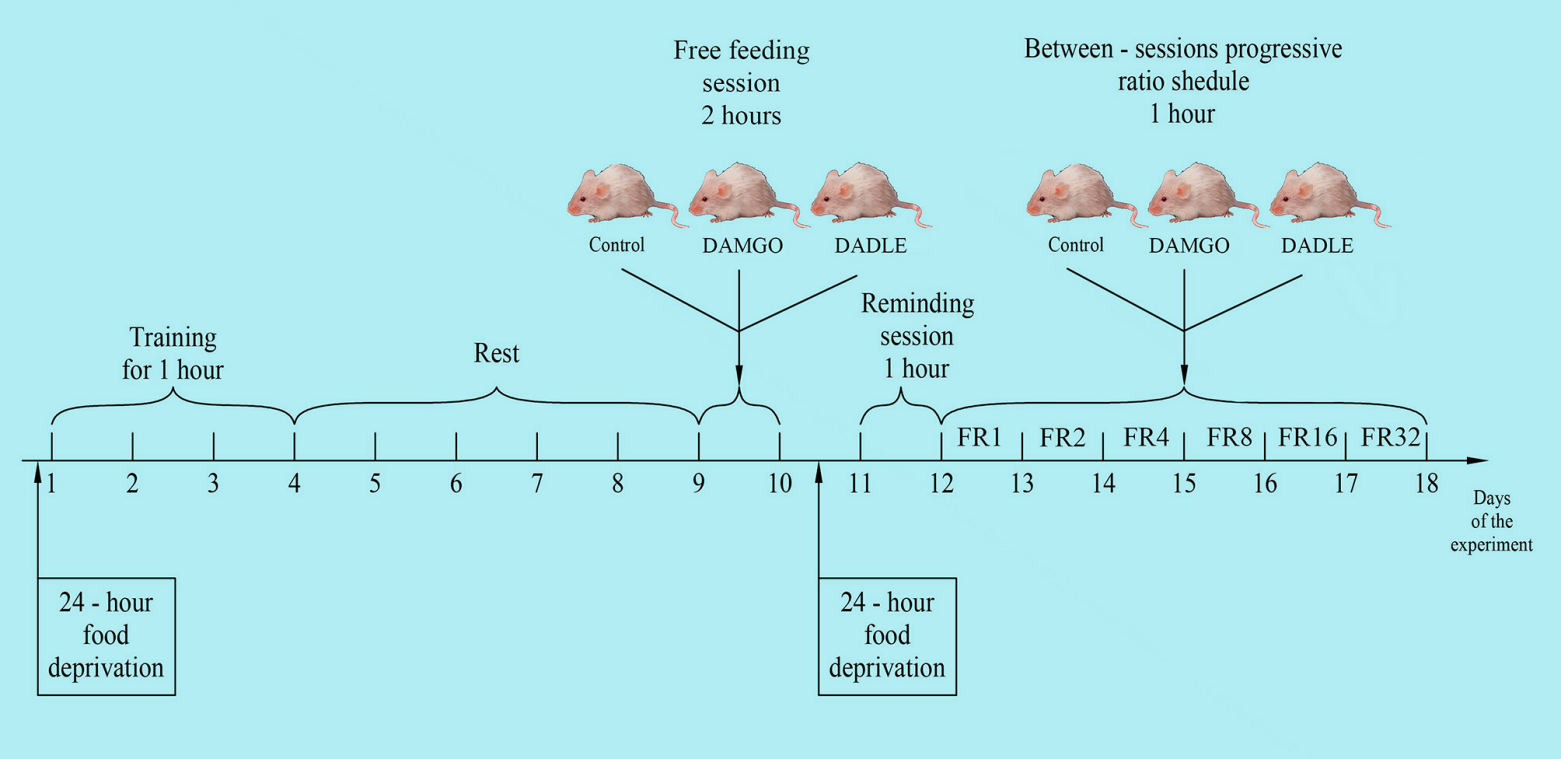
The general scheme of the experiment.

The experiments were performed on 30 Wistar male rats with an average weight of about 230 g before the start of the experiment. The animals were kept in individually ventilated cages of eight animals each, with free access to water and a rat chow (3 kcal/g; Profgryzun, Russia) at a temperature of 21°C in the presence of light from 08:00 to 20:00. All behavioral tests were conducted during the light portion of the cycle. The general scheme of the experiment is shown in Figure 1.

### Training

Experiments were performed using PhenoMaster modular equipment with an integrated operant wall (TSE Systems, Bad Homburg, Germany). One day prior to training, animals were subjected to 24-h food deprivation. Deprivation was carried out to speed up the training process, as we showed earlier (Chumakova et al., 2011; Sudakov et al., 2015). Training was performed in the tool chamber equipped with feeder, lever, and light stimulus. The two metal response levers were located 7.0 cm above the cage floor on either side of the food tray. During lever press training, 1 food pellet (44 mg; 3.6 kcal/g; Bio-Serv®, Flemington, NJ, USA) was presented in a recessed food receptacle after the active lever was pressed. Individual rats were placed in a chamber every 3 days for 1 h. Subsequently, only those animals that produced at least 10 lever pressings within 1 h were chosen to participate in the experiment. Three groups of eight animals were formed from trained animals. Groups were normalized such that the average number of lever pressings from the last training session were equivalent. After training, rats were placed in a home cage with free access to standard feed and water.

### Free Feeding Session

After 5 days the animals were individually placed into PhenoMaster cages in the same environmental conditions as the standard home cages. Animals were recorded every 40 min for 2 h for motor activity by the number of squares crossed, the amount of food eaten and water drunk, as well as oxygen consumption and carbon dioxide production. Thirty minutes before the start of experiments, test solutions were injected directly into the stomach with a special metallic probe in a volume of 0.1 ml per 100 g of body weight. A peptide agonist of mu opioid receptors DAMGO [(D-Ala^2^, *N*-MePhe^4^, Gly-ol)-enkephalin] and a peptide agonist of delta opioid receptors DADLE [(D-Ala^2^, D-Leu^5^)-enkephalin] were used. Peptides rapidly degrade in the gastrointestinal tract and, when introduced directly into the stomach, have no systemic or central effect. The first (control) group of animals (*n* = 10) was administered vehicle alone (water); the second group (*n* = 10) was administered 200 mg/kg DAMGO (Tocris Bioscience, Bristol, UK); and the third group (*n* = 10) was administered 200 mg/kg DADLE (Tocris Bioscience). The dose of the administered substances was selected on the basis of our previous experiments (Trigub et al., 2014; Bogdanova et al., 2015).

### Reminding Session

One day before the start of the next series of experiments, animals were subjected to 24-h food deprivation. On this and following days outside of the 1-h experimental procedure, animals were kept under conditions of limited feeding (8 g of standard feed daily).

On the following day, rats were placed in a modular PhenoMaster unit for a 1 h reminding session where rats received one food pellet after each pressing on the lever. There were no intragastric administrations during the reminding session.

### Between-Sessions Progressive Ratio Schedule

The next day, rats were again placed in experimental cages for 1 h. They received one feed pellet after each pedal press. The number of lever presses to obtain one feed pellet was increased daily to 2, 4, 8, 16, and 32 times during five experimental days, respectively (between-sessions progressive ratio schedule). Thirty minutes before daily placement of the animal in the experimental chamber, the above substances were intragastrically administered. The amount of pellet eaten, feed rate, and locomotor activity were measured. Metabolic rate was calculated by indirect calorimetry by monitoring concentration of oxygen and carbon dioxide in the experimental cages with electrochemical sensors. All parameters were measured at 15 min intervals for 1 h.

### Determination of The Level of Food Motivation

The rate of response shows the number of feed pellets eaten per time interval and reflects the magnitude of the food motivation of the animal. Thus, the level of food motivation can be estimated by the number of lever presses to receive food at each 15 min timepoint of the experiment. The more intense the rate of response, the higher the level of motivation. So, if the motivational level of a hungry animal is normalized to 100%, then under the condition of fixed ratio (FR) 1, in 45 min the rats completely satisfy the food motivation. In the last 15 min of the hourly session, most rats have never pressed the lever and the level of food motivation can be considered equal to zero (Figure 1). Intermediate states can be expressed as a percentage of the initial motivation according to the formula: M15 = 100% – (P15/Ptotal ^*^ 100%) (for the level of motivation after 15 min of eating behavior); M30 = 100% – (P15 + P30/Ptotal ^*^ 100%) (for the level of motivation after 30 min of eating behavior); M45 = 100% – (P15 + P30 + P45/Ptotal ^*^ 100%) (for the level of motivation after 45 min of eating behavior); and M60 = 100% – (P15 + P30 + P45 + P60/Ptotal ^*^ 100%) (for the level of motivation after 60 min of eating behavior). For these calculations, M is the level of motivation, P15, P30, P45, and P60 are the number of food pellets eaten for the corresponding period of time (0–15, 15–30, 30–45, or 45–60 min, respectively), and Ptotal is the total amount of eaten granules necessary for full satiation. In general, Ptotal during FR 1 averaged 152 ± 25 granules. Since rats on different FR reinforcement schedules needed to pay different energy costs for food-producing behavior, animals could not fully satisfy food motivation during the hour-long experiment and the number of pellets eaten could not accurately reflect the magnitude of motivation. The level of motivation can be very high whereas the number of pellets eaten can be small due to the large energy costs for their procurement. In this regard, we used a correction coefficient which reflected the activating effect of food motivation on metabolism (Sudakov et al., 2015). The coefficient is equal to the current energy consumption in kcal/h/kg divided by the average basal level of rat metabolism in each corresponding time interval. The average level of Wistar rat metabolism according to our previous experiments (Sudakov and Bashkatova, 2013; Sudakov et al., 2017) is 6.0 kcal/h/kg. Thus, for an animal in conditions of limited feeding, at the beginning of instrumental eating behavior, the level of food motivation can be adjusted to 100%, after *n* minutes it can be calculated as Mn ^*^ kn, where kn is the metabolic coefficient determined for the interval 0–15, 15–30, 30–45, or 45–60 min.

### Statistics

Since the measured variable did not meet normality, non-parametric statistical methods were applied. The Kruskal–Wallis test showed that the mean ranks of the groups were not equal. As a result, two groups comparison was applied using the Mann–Whitney U test. The results were considered significant at *p* < 0.05. All results are presented as mean ± standard deviation.

### Ethical Statement

The protocols and procedures for this study were ethically reviewed and approved by the Animal Care and Use Committee of the P.K. Anokhin Research Institute of Normal Physiology (Permission number 328) and conform to Directive 2010/63/EU.

## Results

### Free Feeding Session

It was found that the administration of neither DAMGO nor DADLE affects eating behavior, motor activity, and metabolism of well-fed rats with free access to food (Table 1).

**Table 1:** The total amount of food eaten, motor activity, and average metabolism during 2 h of free access to food.

	**Food eaten (pellets)**	**Motor activity (units)**	**Metabolism ( kkal/h/kg)**
Control	18.9 ± 1.6	3290 ± 115.4	6.79 ± 0.6
DAMGO	25.0 ± 1.8	3624 ± 121.7	7.05 ± 0.7
DADLE	24.7 ± 1.7	2174 ± 110.1	6.63 ± 0.5

### Between-Sessions Progressive Ratio Schedule

**Figure 2:**
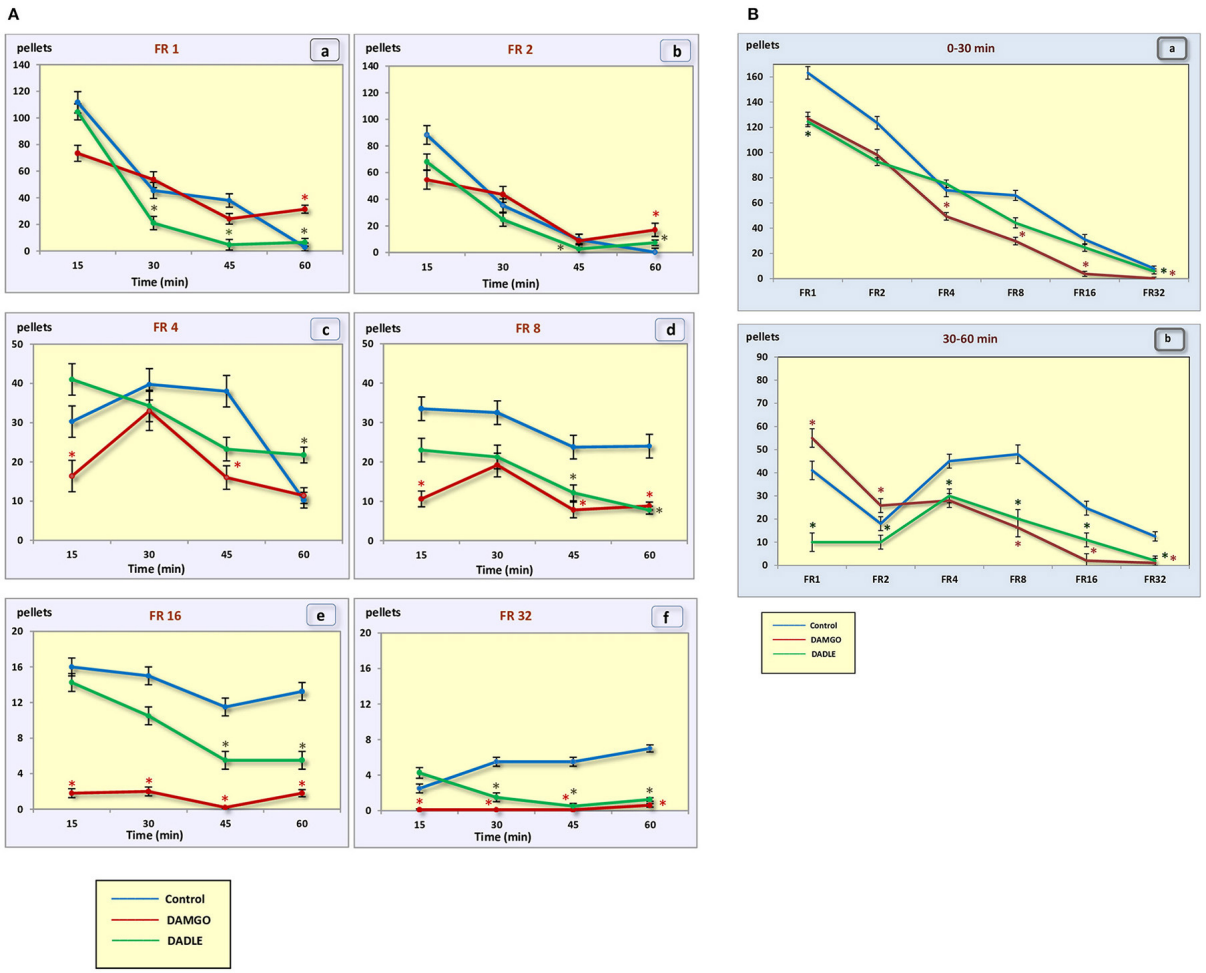
**(A)** Number of pellets eaten during a 1 h session. (a) – FR 1 reinforcement schedule; (b) – FR 2 reinforcement schedule; (c) – FR 4 reinforcement schedule; (d) – FR 8 reinforcement schedule; (e) – FR 16 reinforcement schedule; (f) – FR 32 reinforcement schedule; *p < 0.05 compared to control. **(B)** Number of pellets eaten during 0–30 min (a) and 30–60 min (b) of 1 h session. *p < 0.05 compared to control.

In the control group of hungry, trained rats, when the fixed ratio reinforcement schedule was FR 1 and FR 2, approximately the same rates of response and dynamics of lever pressing were observed. In this regard, the rats were able to eat almost two times less food when the fixed ratio reinforcement schedule became FR 2 (Figures 2A,B). At the beginning of the experimental session, the rate of response was 7.6 and 12 times per minute, respectively. Then the rate gradually decreased, reaching two and three presses per minute by 45 min, respectively, and by 60 min it was near 0. For the FR 4 reinforcement schedule (medium difficulty), the initial rate of 10.5 lever presses remained practically unchanged for 45 min, and it decreased to 3.8 presses per minute only in the last 15 min. With FR 8, the initial rate of response was 19.5 per minute, and in the last 15 min it decreased to 15.6 presses per minute. The same initial rate was observed for FR 16, however, by the end of the session the rate of response did not decrease, and even increased slightly, reaching an average value of 20.8. With a “high difficulty” in food-producing behavior at FR 32, the rat's rate of response increased throughout the session.

In the “light” variants of operant feeding behavior (FR 1, FR 2), the high food motivation at the beginning of the session gradually decreased over the course of an hour, and by the end of the session the animals were completely satiated.

**Figure 3:**
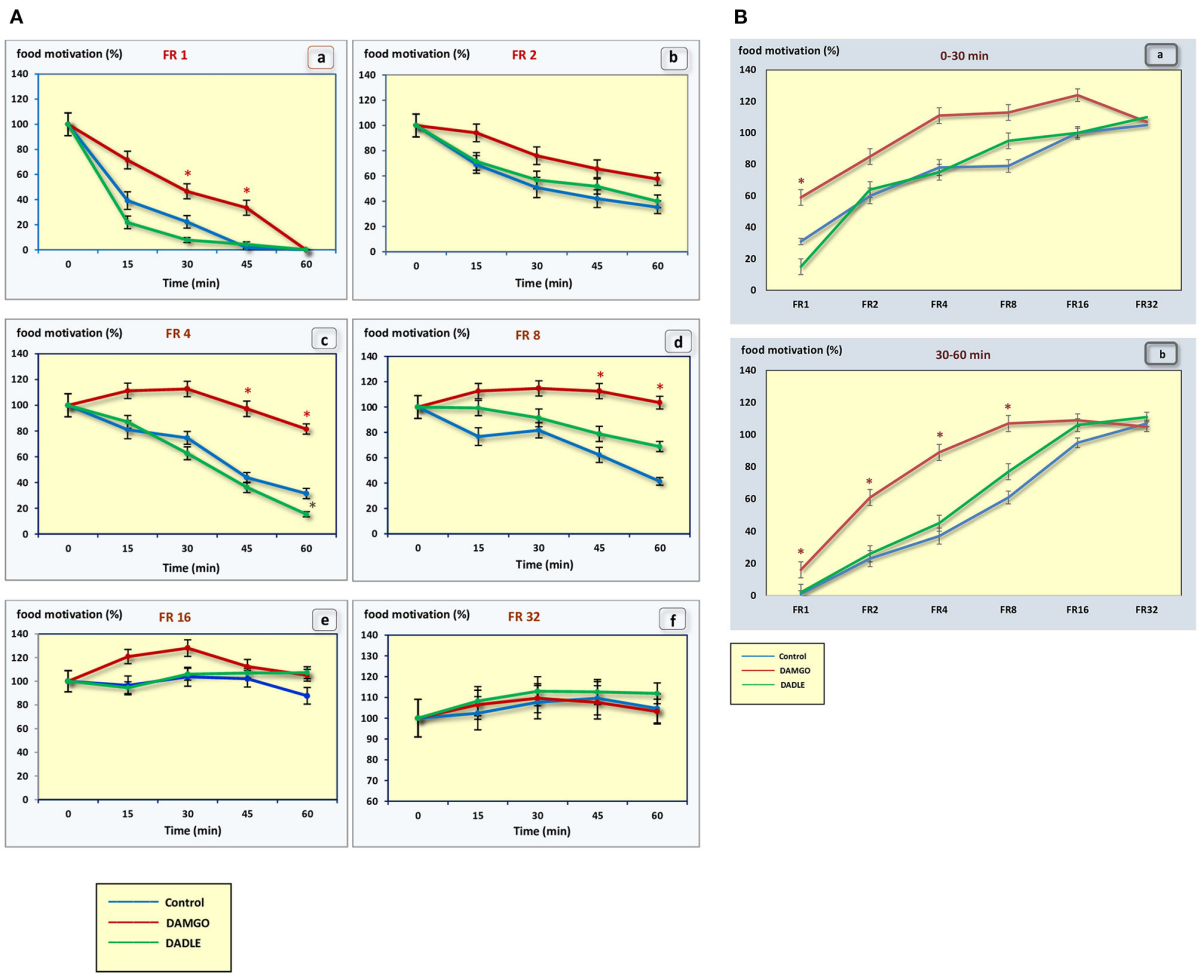
**(A)** The level of food motivation as a percentage of the basal level during a 1 h session. (a) – FR 1 reinforcement schedule; (b) – FR 2 reinforcement schedule; (c) – FR 4 reinforcement schedule; (d) – FR 8 reinforcement schedule; (e) – FR 16 reinforcement schedule; (f) – FR 32 reinforcement schedule; *p < 0.05 compared to control. **(B)** The level of food motivation as a percentage of the basal level during 0–30 min (a) and 30–60 min (b) of 1 h session. *p < 0.05 compared to control.

With an increase in difficulty for obtaining food, at the beginning of the experiment, food motivation remains at approximately the same level, and then begins to increase (Figures 3A,B). The effectiveness of operant feeding behavior as the number of pellets eaten decreases with increasing complexity (Figure 2).

**Figure 4:**
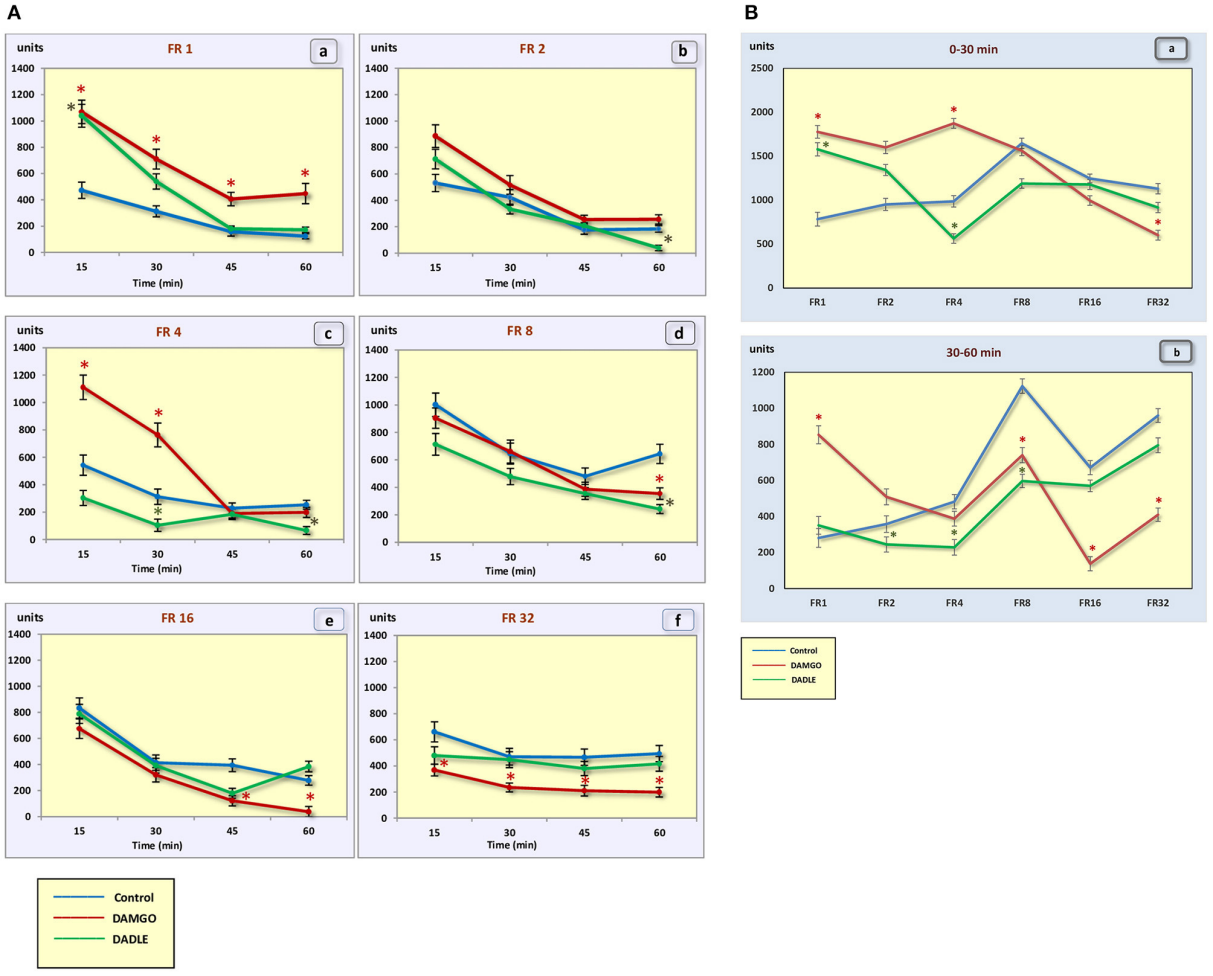
**(A)** The motor activity (square crossings) during 1 h sessions. (a) – FR 1 reinforcement schedule; (b) – FR 2 reinforcement schedule; (c) – FR 4 reinforcement schedule; (d) – FR 8 reinforcement schedule; (e) – FR 16 reinforcement schedule; (f) – FR 32 reinforcement schedule; *p < 0.05 compared to control. **(B)** The motor activity (square crossings) during 0–30 min (a) and 30–60 min (b) of 1 h session. *p < 0.05 compared to control.

Intragastric administration of a mu opioid receptor agonist DAMGO led to an increase in the level of food motivation in “light” operant feeding behaviors (FR 1, FR 4, and FR 8) (Figure 3Aa, c, d). Most of differences were obtained in the second half of the sessions (Figure 3B; Table 2). Feed consumption did not change (Figure 2Aa,b; Table 1). At high costs for feeding behavior (FR 16, FR 32), administration of DAMGO did not lead to a change in food motivation (Figure 3Ae,f; Table 2), however feed consumption and motor activity were reduced (Figure 2Ac–f; Figure 2Bb; Figure 4Ae, f; Figure 4Bb; Table 2). Significant differences were observed throughout the experiment (Table 2).

**Table 2:** Statistical analysis data.

		**15 min**	**30 min**	**45 min**	**60 min**
Food eaten	FR1	*U* = 22.00, *Z* = 0.99, *p* = 0.328	*U* = 23.00, *Z* = 0.63, *p* = 0,505	*U* = 23.00, *Z* = 0.57, *p* = 0.573	*U* = 8.00, *Z* = 2.72, *p* = 0.002
	FR2	*U* = 25.00, *Z* = 0.50, *p* = 0.645	*U* = 22.00, *Z* = 0.88, *p* = 0.382	*U* = 22.00, *Z* = 0.68, *p* = 0.541	*U* = 8.00, *Z* = 2.76, *p* = 0.001
	FR4	*U* = 10.00, *Z* = 2.50, *p* = 0.011	*U* = 23.00, *Z* = 0.82, *p* = 0.452	*U* = 8.00, *Z* = 2.74, *p* = 0.001	*U* = 24.00, *Z* = 0.50, *p* = 0.696
	FR8	*U* = 13.00, *Z* = 2.04, *p* = 0.035	*U* = 19.00, *Z* = 1.14, *p* = 0.244	*U* = 10.00, *Z* = 2.52, *p* = 0.011	*U* = 10.0, *Z* = 2.25, *p* = 0.017
	FR16	*U* = 8.00, *Z* = 2.76, *p* = 0.001	*U* = 8.00, *Z* = 2.72, *p* = 0.002	*U* = 8.00, *Z* = 2.74, *p* = 0.001	*U* = 8.00, *Z* = 2.76, *p* = 0.001
	FR32	*U* = 7.50, *Z* = 2.78, *p* = 0.001	*U* = 7.50, *Z* = 2.76, *p* = 0.001	*U* = 7.50, *Z* = 2.78, *p* = 0.001	*U* = 7.50, *Z* = 2.78, *p* = 0.001
Food motivation	FR1	*U* = 15.00, *Z* = 2.42, *p* = 0.059	*U* = 13.00, *Z* = 2.08, *p* = 0.037	*U* = 8.00, *Z* = 2.71, *p* = 0.002	*U* = 20.50, *Z* = 0.88, *p* = 0.374
	FR2	*U* = 21.00, *Z* = 0.84, *p* = 0.378	*U* = 21.00, *Z* = 0.92, *p* = 0.364	*U* = 18.00, *Z* = 1.18, *p* = 0.237	*U* = 18.00, *Z* = 1.19, *p* = 0.233
	FR4	*U* = 19.00, *Z* = 1.06, *p* = 0.289	*U* = 19.00, *Z* = 1.12, *p* = 0.257	*U* = 10.00, *Z* = 2.24, *p* = 0.012	*U* = 10.00, *Z* = 2.36, *p* = 0.011
	FR8	*U* = 18.00, *Z* = 1.56, *p* = 0.163	*U* = 18.00, *Z* = 1.44, *p* = 0.189	*U* = 13.00, *Z* = 1.96, *p* = 0.048	*U* = 13.00, *Z* = 2.06, *p* = 0.033
	FR16	*U* = 20.00, *Z* = 0.98, *p* = 0.325	*U* = 20.50, *Z* = 0.90, *p* = 0.347	*U* = 20.00, *Z* = 1.01, p = 0.310	*U* = 19.00, *Z* = 1.10, *p* = 0.267
	FR32	*U* = 22.00, *Z* = 0.68, *p* = 0.543	*U* = 23.00, *Z* = 0.52, *p* = 0.674	*U* = 25.00, *Z* = 0.34, *p* = 0.731	*U* = 27.00, *Z* = 0.25, *p* = 0.754
Motor activity	FR1	*U* = 11.00, *Z* = 2.20, *p* = 0.021	*U* = 11.00, *Z* = 2.00, *p* = 0.044	*U* = 11.00, *Z* = 2.10, *p* = 0.032	*U* = 8.00, *Z* = 2.76, *p* = 0.001
	FR2	*U* = 18.00, Z = 1.18, *p* = 0.238	*U* = 22.00, Z = 0.72, *p* = 0.448	*U* = 22.00, *Z* = 0.70, *p* = 0.460	*U* = 22.00, *Z* = 0.68, *p* = 0.520
	FR4	*U* = 11.00, *Z* = 2.08, *p* = 0.036	*U* = 8.00, *Z* = 2.60, *p* = 0.005	*U* = 21.00, *Z* = 0.88, *p* = 0.376	*U* = 21.00, *Z* = 0.86, *p* = 0.348
	FR8	*U* = 22.00, *Z* = 0.86, *p* = 0.416	*U* = 23.00, *Z* = 0.80, *p* = 0.488	*U* = 22.00, *Z* = 0.86, *p* = 0.420	*U* = 10.00, *Z* = 2.00, *p* = 0.043
	FR16	*U* = 21.00, *Z* = 1.04, *p* = 0.314	*U* = 22.00, *Z* = 0.88, *p* = 0.386	*U* = 11.00, *Z* = 2.04, *p* = 0.041	*U* = 8.00, *Z* = 2.64, *p* = 0.003
	FR32	*U* = 8.00, *Z* = 2.72, *p* = 0.002	*U* = 8.00, *Z* = 2.70, *p* = 0.002	*U* = 8.00, *Z* = 2.76, *p* = 0.001	*U* = 8.00, *Z* = 2.78, *p* = 0.001

*Comparison of food eaten, motivation level and motor activity between control and DAMGO groups*.

Intragastric administration of delta opioid receptors agonist DADLE did not lead to changes in levels of feeding motivation and physical activity, but inhibition of feeding behavior was observed for all reinforcement schedules (Figure 2A). Significant changes were observed, mainly in the second half of the hourly session (Figure 2B; Table 3).

**Table 3:** Statistical analysis data.

	**15 min**	**30 min**	**45 min**	**60 min**
FR1	*U* = 17.00, *Z* = 1.62, *p* = 0.132	*U* = 11.00, *Z* = 2.20, *p* = 0.024	*U* = 8.00, *Z* = 2.74, *p* = 0.001	*U* = 10.00, *Z* = 2.46, *p* = 0.011
FR2	*U* = 18.00, *Z* = 1.12, *p* = 0.256	*U* = 19.00, *Z* = 1.10, *p* = 0.274	*U* = 11.00, *Z* = 2.25, *p* = 0.022	*U* = 8.00, *Z* = 2.76, *p* = 0.001
FR4	*U* = 18.00, *Z* = 1.14, p = 0.244	*U* = 18.00, *Z* = 1.18, *p* = 0.212	*U* = 19.00, *Z* = 1.06, *p* = 0.296	*U* = 11.00, *Z* = 2.15, *p* = 0.027
FR8	*U* = 19.00, *Z* = 1.02, *p* = 0.302	*U* = 20.00, *Z* = 1.04, *p* = 0.310	*U* = 11.00, Z = 2.25, *p* = 0.022	*U* = 10.00, *Z* = 2.32, *p* = 0.016
FR16	*U* = 18.00, *Z* = 1.14, *p* = 0.248	*U* = 17.00, *Z* = 1.58, *p* = 0.144	*U* = 10.00, *Z* = 2.34, p = 0.015	*U* = 10.00, *Z* = 2.36, *p* = 0.014
FR32	*U* = 17.00, *Z* = 1.64, *p* = 0.130	*U* = 8.00, *Z* = 2.70, *p* = 0.002	*U* = 8.00, *Z* = 2.74, *p* = 0.001	*U* = 8.00, *Z* = 2.76, *p* = 0.001

*Comparison of food eaten between control and DADLE groups*.

**Figure 5:**
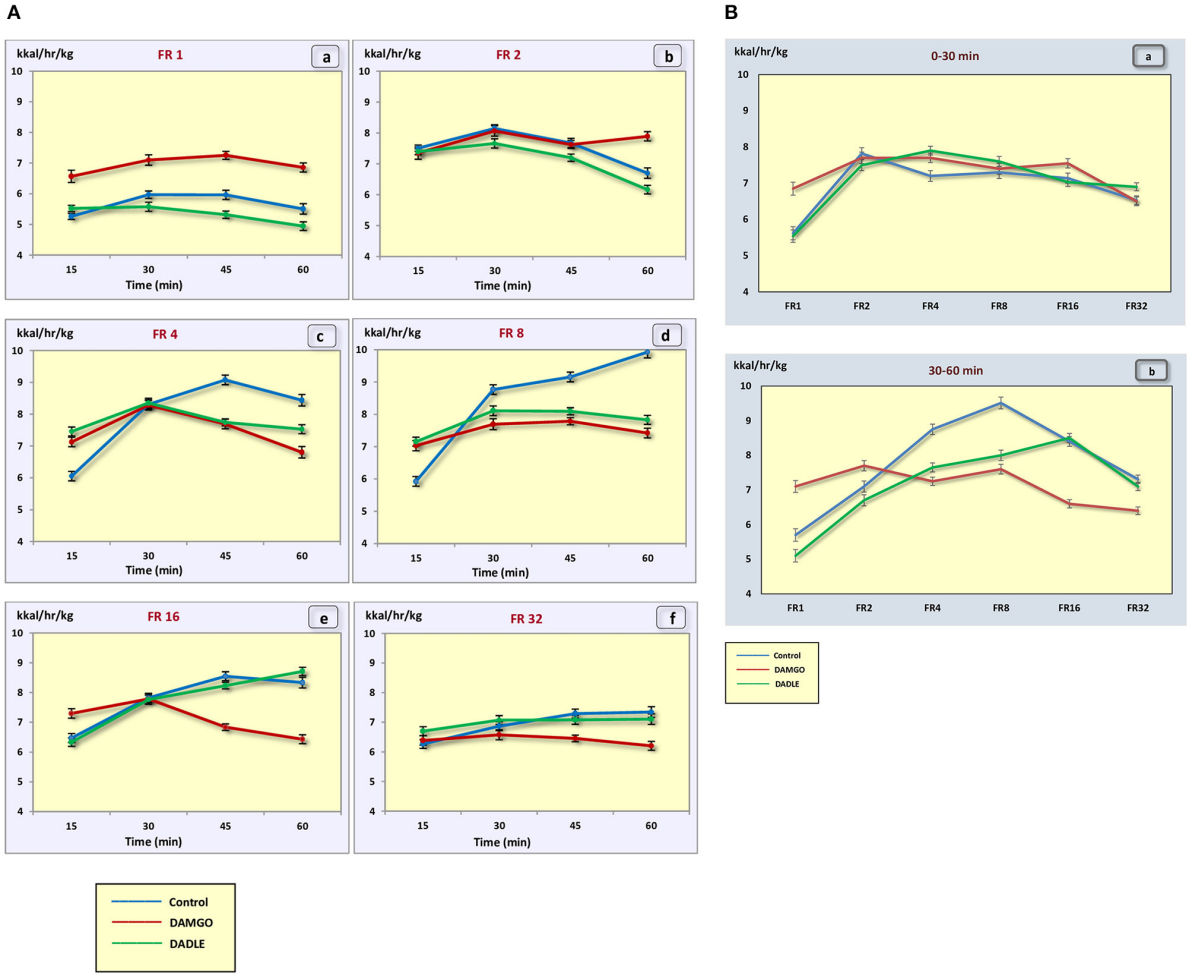
**(A)** The metabolic rate (kcal/h/kg) during 1 h sessions. (a) – FR 1 reinforcement schedule; (b) – FR 2 reinforcement schedule; (c) – FR 4 reinforcement schedule; (d) – FR 8 reinforcement schedule; (e) – FR 16 reinforcement schedule; (f) – FR 32 reinforcement schedule; *p < 0.05 compared to control. **(B)** The metabolic rate (kcal/h/kg) during 0–30 min (a) and 30–60 min (b) of 1 h session. *p < 0.05 compared to control.

The administration of neither DAMGO nor DADLE led to changes in the level of animal metabolism during instrumental eating behavior (Figures 5A,B).

## Discussion

Our results showed significant differences in the mechanisms of implementation of food motivation in behavior, which depended upon the conditions for achieving the result. With free access to food and weak food motivation, gastric opioid receptors were not involved in regulation of feeding behavior. However, their activation can significantly change the processes of organizing eating behavior in the presence of increasing levels of food motivation and energy costs to satisfy it. The same data was obtained earlier with the introduction of DAMGO into the subthalamic nucleus. DAMGO microinfusions had no effect on FR 2 performance. Nevertheless, mu opioid receptor stimulation significantly increased feeding on a palatable diet and reduced the reinforcers earned on a DRL20 schedule (Pratt et al., 2012). We observed the opposite effect. The introduction of DAMGO into the stomach led to a suppression of food intake. This is fully consistent with our hypothesis of a reciprocal relationship between the central and peripheral opioid systems (Sudakov and Trigub, 2008). Earlier, Levine and colleagues demonstrated that involvement of the opioid system in feeding behavior may depend on the level of motivation. The efficacy of naloxone at reducing food intake was inversely related to the level of food deprivation that the animal was subjected to Rudski et al. (1994), Levine et al. (1995), Weldon et al. (1996).

The disappearance of the DAMGO effect on days 5 and 6 outwardly looks like the development of tolerance. In fact, an increase in the effect of DAMGO on feed intake is observed precisely on days 5 and 6. However, it is possible, but unlikely, that on the 5th and 6th day of DAMGO administration, tolerance to the effect on motivation and sensitization to the effect on food intake develops. Additional experiments are needed to verify this assumption.

**Figure 6:**
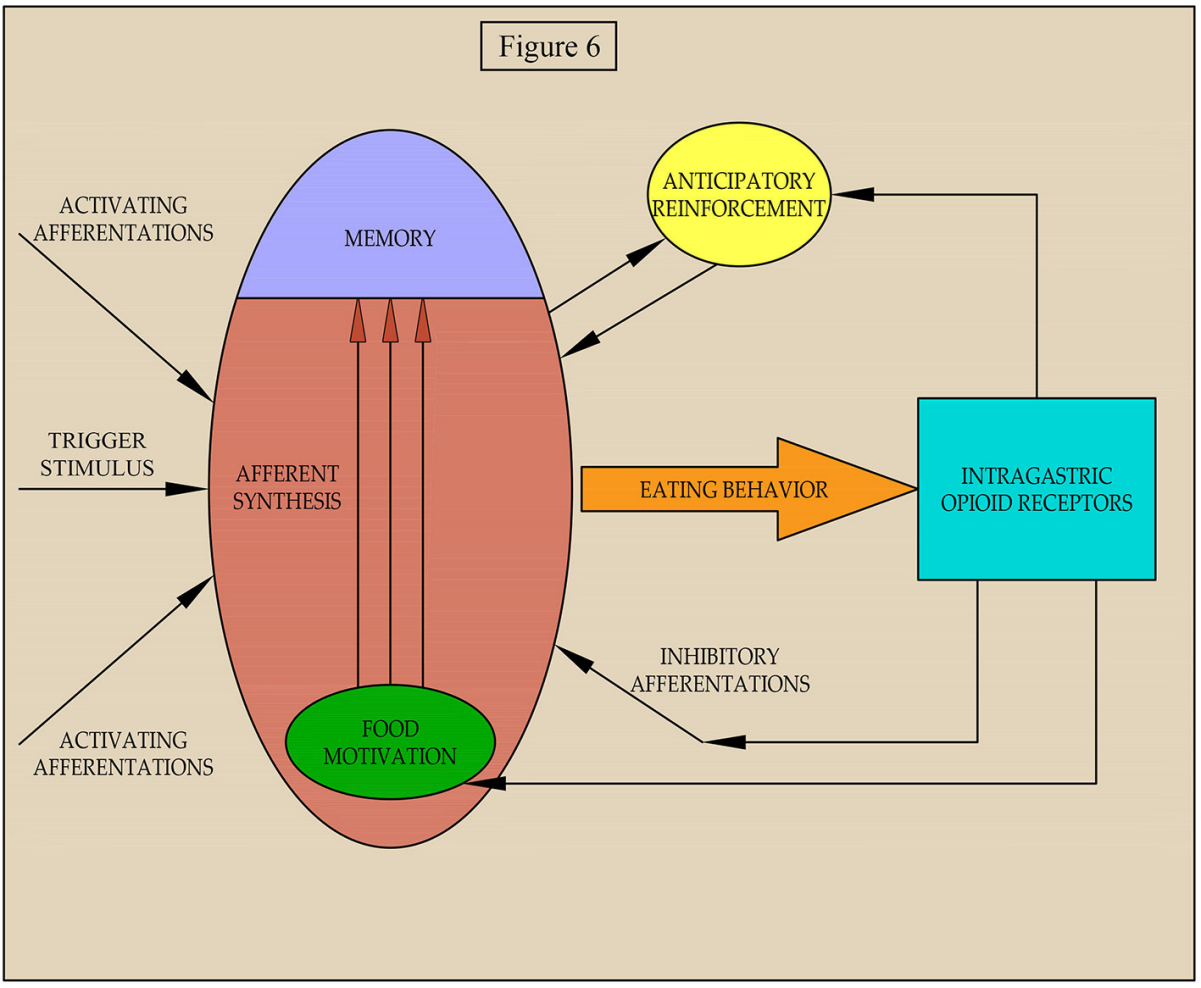
Scheme of regulation of the implementation of food motivation into eating behavior.

According to the theory of functional systems by P.K. Anokhin (Anokhin and Serzhantov, 1973), nutritional needs form a motivation that, at the stage of afferent synthesis, extracts from the memory genetic and individually acquired information about the program of eating behavior that is optimal in a particular environment. The decision to start eating behavior is based on the integration of motivational arousal, memory, and afferent situational arousal, which can either activate the decision or slow it down (Figure 6).

Our data suggest that afferentation from gastric receptors can affect both the level of food motivation and the processes of its implementation in food behavior whenever there is a pronounced food motivation that requires some effort to satisfy. It is possible that both in our experiment and in a natural setting for eating of food, the activation of mu opioid receptors in the stomach by peptides with opioid activity leads to simultaneous increases of inhibitory afferentations food motivation. This balances the impact on afferent synthesis and eating behavior and is not affected by weak inhibitory afferentations. With further activation of the inhibitory afferentations, despite increased motivation, eating behavior is suppressed.

As our earlier data suggests, activation of mu opioid receptors of the stomach leads to vagal afferentation (Sudakov et al., 2012) causing a decrease in release of beta-endorphin and reducing the affinity of mu opioid receptors in the midbrain and cortex (Proskuryakova et al., 2009; Sudakov et al., 2010). It is known that nutritional reinforcement is due to the activation of the mesocorticolimbic dopamine system, which occurs with the direct participation of beta-endorphin in the ventral tegmentum area. If the amount of beta-endorphin is decreased by activation of the gastric mu opioid receptor, then food reinforcement should change. We propose a two-stage mechanism of positive reinforcement. The first stage, anticipatory reinforcement, is formed when the result is still not achieved. At this stage, the importance of the planned result and the probability of its achievement are assessed. The greater these indices are, the stronger the anticipatory reinforcement becomes. Hypothetically, anticipatory reinforcement is mediated by dopamine release from nerve terminals in the mesencephalon (Sudakov, 2019). If the likelihood of achieving a result decreases, for example, with an increase in energy expenditure for eating behavior, the intensity of anticipatory reinforcement will also decrease. Since, anticipatory reinforcement stimulates the implementation of food motivation in behavior, when it is suppressed, the behavior will also be inhibited.

Thus, we hypothesize that central and peripheral mechanisms are involved in regulating the implementation of food motivation into eating behavior under conditions of varying difficulty to achieve a result. With poor food motivation and free access to food, the peripheral regulation mechanism is not involved.

We propose three pathways of regulation of eating behavior in difficult food conditions by gastric opioid receptors: (I) environmental-inhibitory afferentations and suppression of the implementation of food motivation in behavior; (II) homeostatic-inhibitory action on food motivation; and (III) rewarding-suppression of the anticipatory reinforcement. We hypothesize that excitation from opioid receptors in the stomach is transmitted through vagal afferentation to the nucleus tractus solitaries. There are anatomical evidence shows mRNA expression of serotonin receptors on GLP-1-producing preproglucagon (PPG) neurons in the medial nucleus tractus solitarius by fluorescent *in situ* hybridization, suggesting that PPG neurons are likely to express these receptors (Leon et al., 2019). GLP-1 is an anorectic hormone involved in the control of food intake (Barrera et al., 2011). Thus, the first pathway can be realized through the release of serotonin and glucagon in hindbrain, which can cause hypophagia. The second pathway follows from the well-known facts about suppression of food motivation when food enters the stomach. This causes the release of glucose from the depot and the effect on glucose-sensitive neurons of the hypothalamus (Balagura and Kanner, 1971; Oomura, 1981). The third pathway is based upon data from a number of studies on mechanisms of reward (Majeed et al., 1986; Bakshi and Kelley, 1993; Kelley et al., 1996; Zhang et al., 1998) and anticipation (Barbano and Cador, 2006) and the participation of opioids in the mesocorticolimbic system of the brain, and our early studies showing the possibility of gastric opioid receptors acting on these central processes (Proskuryakova et al., 2009; Sudakov et al., 2010, 2012; Sudakov, 2019). Of course, the above neurochemical mechanisms of the effect of opioid receptors on eating behavior are still speculative and will be the subject of our further research.

Thus, our data indicates experimental confirmation of the mechanism of peripheral regulation of the implementation of food motivation in behavior. This result opens up the possibility for influencing this process with relatively safe peripherally acting drugs, nutritional supplements, and foods with opioid activity.

## Data Availability Statement

The raw data supporting the conclusions of this article will be made available by the authors, without undue reservation.

## Ethics Statement

The animal study was reviewed and approved by Animal Care and Use Committee of the P.K. Anokhin Research Institute of Normal Physiology (Permission number 328).

## Author Contributions

SS wrote the article. NB processed the data and carried out the technical design of the article. All authors have read and agreed to the published version of the manuscript and contributed equally to the data collection for this work.

## Conflict of Interest

The authors declare that the research was conducted in the absence of any commercial or financial relationships that could be construed as a potential conflict of interest.
